# An experimental data library for the full CsPb(Cl_*x*_Br_1−*x*_)_3_ compositional series†

**DOI:** 10.1039/d5cc00735f

**Published:** 2025-03-31

**Authors:** Kinga O. Mastej, Bodoo Batnaran, Antti-Pekka M. Reponen, Zachary A. VanOrman, Kal Banger, Michael A. Hayward, Volker L. Deringer, Sascha Feldmann

**Affiliations:** a Rowland Institute, Harvard University Cambridge USA; b Inorganic Chemistry Laboratory, Department of Chemistry, University of Oxford Oxford UK volker.deringer@chem.ox.ac.uk; c Institute of Chemical Sciences and Engineering, Ecole Polytechnique Fédérale de Lausanne Lausanne, Switzerland sascha.feldmann@epfl.ch

## Abstract

A complete series of CsPb(Cl_*x*_Br_1−*x*_)_3_ mixed-halide perovskites with *x* = 0–1 in small steps is reported, and their structural and optical properties characterised. A comparison of synthetic approaches shows that mechanosynthesis yields the most robust data across the compositions, avoiding solvent inclusion or miscibility gaps. The resulting data library, including some hitherto unreported compositions, can serve as a benchmark for future computational modelling.

## Abstract 2

A complete series of CsPb(Cl_*x*_Br_1−*x*_)_3_ mixed-halide perovskites with *x* = 0–1 in small steps is reported, and their structural and optical properties characterised. All data is accessible in a machine-readable format.

## Graphical abstract

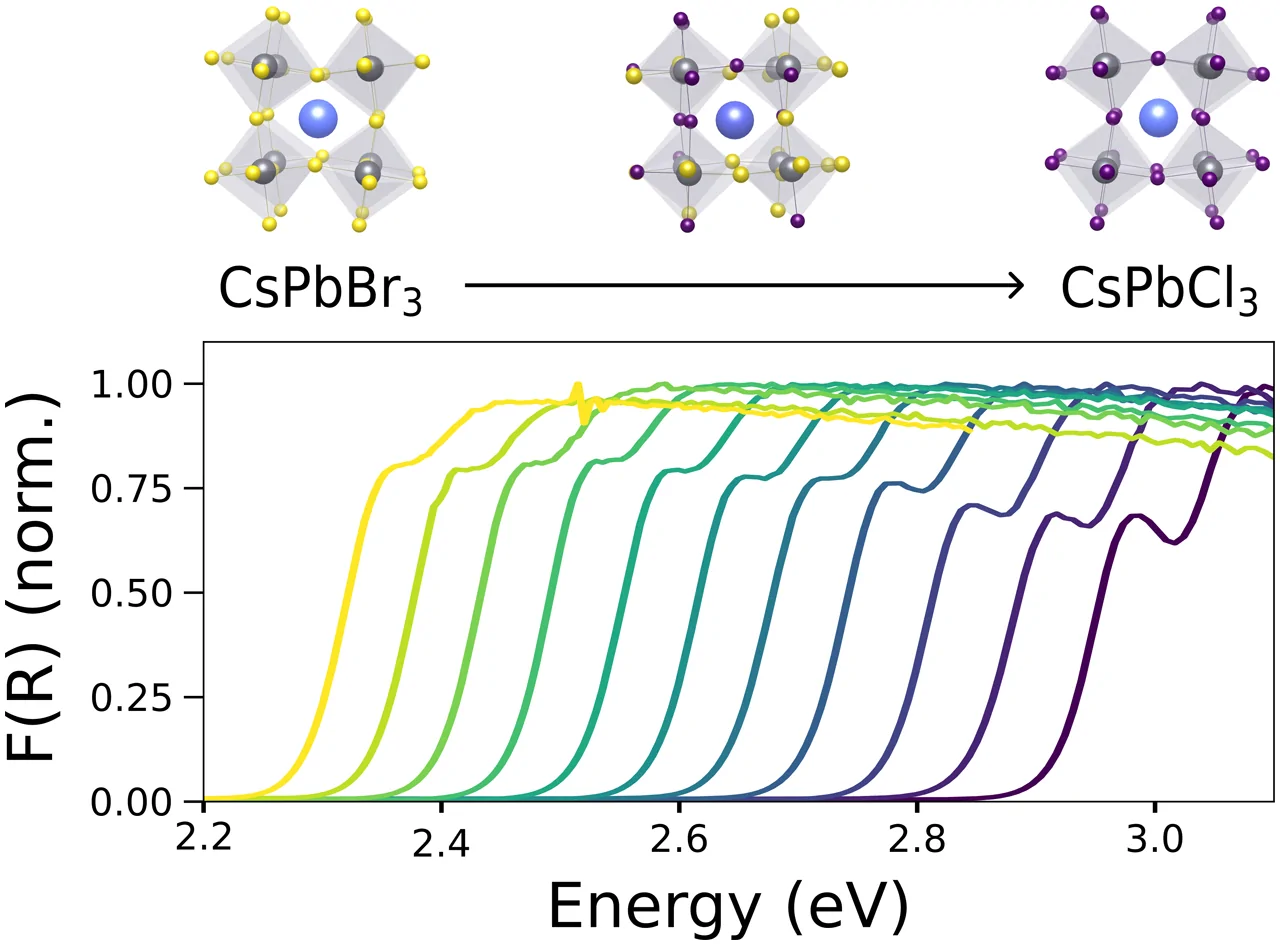

Halide perovskites have attracted widespread attention due to their outstanding optoelectronic properties, such as high optical absorption coefficients,^1^ high charge carrier mobility,^2^ and long carrier lifetimes.^3^ These properties make them promising for efficient thin-film solar cells and light-emitting applications.^4–6^ Such applications are largely dependent on the successful tuning of their properties to obtain the desired optical and electrical performance. The versatility of the perovskite structure (Fig. 1a) offers a rich chemical space to explore the desired property targets,^7^ for example, by changing the halide composition and mixing halide anions to tune the optical band gap.^8^ Simultaneously, the efficiency of exploring this compositional search space is aided by new theoretical approaches, such as interatomic potentials machine-learned from quantum-mechanical reference data.^9^ As these models infer the nature of atomic interactions exclusively from computed datasets, direct validation against experimental data is the ultimate test for a machine-learned potential.^10^

**Fig. 1:**
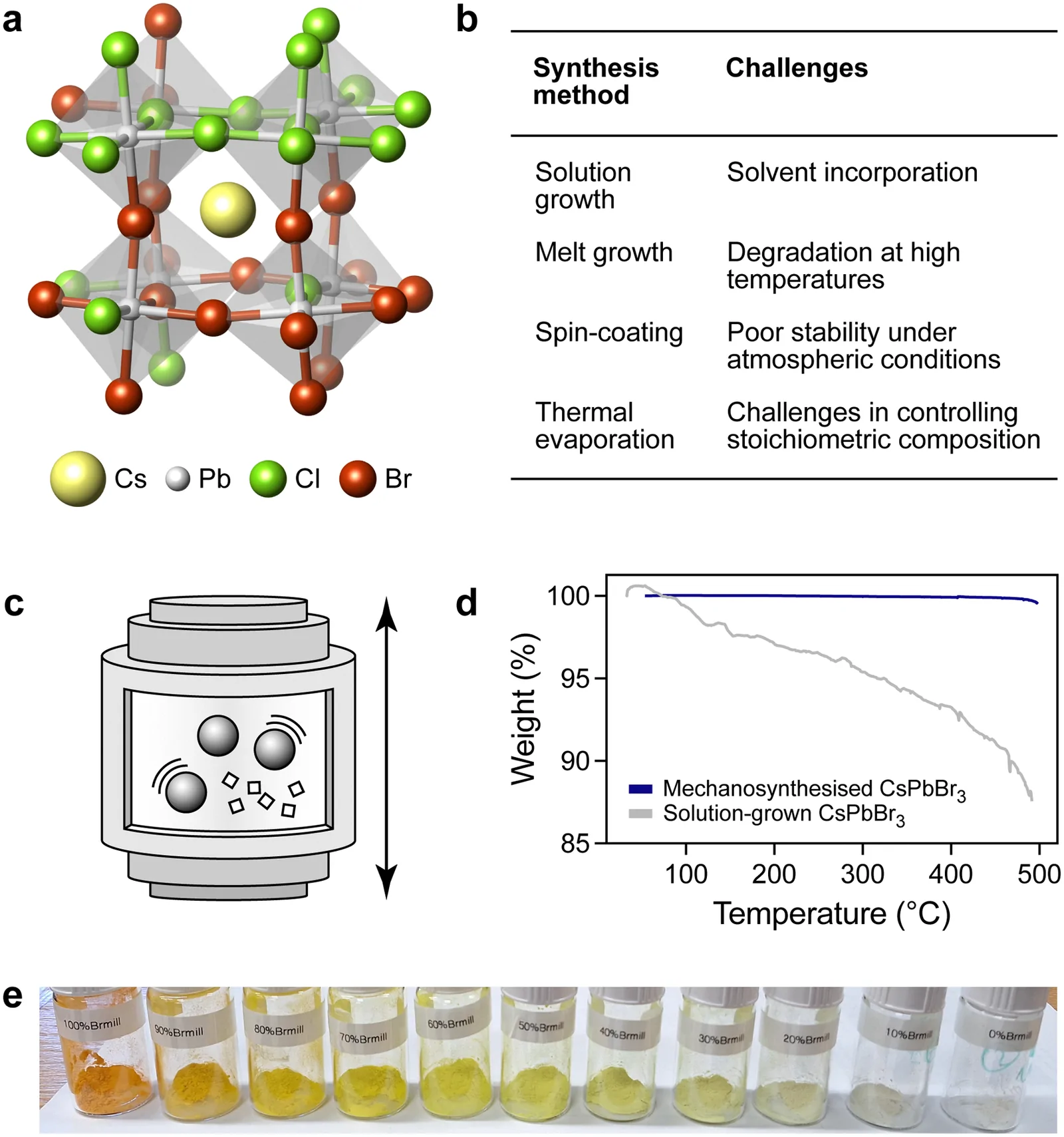
(a) Fragment from a special quasirandom structure^11^ (SQS) model created for CsPbCl_1.5_Br_1.5_ composition based on the structural data for ICSD 143611^12^ in ICET.^13^ Image produced using VESTA.^14^ (b) Comparison of alternative synthetic methods for accessing halide perovskite materials with their respective key difficulties – solvent incorporation,^15^ thermal degradation,^16^ poor stability^17^ and challenging stoichiometric control.^18^ (c) Schematic of ball milling used in the mechanosynthesis of halide perovskites. (d) TGA comparison of mechanosynthesised and solution-grown CsPbBr_3_. Weight loss indicates solvent incorporation in the solution-grown crystals, making them less suitable samples. At the beginning of a TGA experiment, the rising temperature decreases gas density, resulting in a lower buoyant force, and small apparent, artificial mass gain reading.^19^ (e) Photograph of mechanosynthetically prepared CsPb(Cl_*x*_Br_1−*x*_)_3_ powder series.

However, computational modelling of this class of materials is limited by incomplete, inconsistent, or poorly reproducible experimental reference data.^20^ For example, for the fully inorganic and lighter element-containing prototype composition CsPb(Cl_*x*_Br_1−*x*_)_3_, which would act as the starting point for many computational studies, various publications exist,^21,22^ each typically focusing on a subset of compositions, and on specific experimental tools relevant to that particular study. Accordingly, trends between different compositions are difficult to directly assess across these reports. Early studies typically report numerical data in the form of image files, making it cumbersome to read them out. Crucially, the Crystallographic Information Files (CIFs) of mixed-composition CsPb(Cl_*x*_Br_1−*x*_)_3_ phases are not to be found in online databases such as COD,^23^ ICSD,^24^ The Materials Project^25^ or any other, to our best knowledge. This absence hinders the atomistic modelling of this family of materials and high-throughput computational studies.

To fill this gap, a complete compositional series of CsPb(Cl_*x*_Br_1−*x*_)_3_ mixed-halide perovskites was synthesised using a mechanochemical approach as compared to the more extensively studied thin films, which display high levels of heterogeneity between the samples,^26^ leading to inconsistencies between the data published even within the same laboratory.^27^ The series was characterised by X-ray diffraction (XRD), thermogravimetric analysis (TGA), optical reflectance and photoluminescence (PL) measurements. The goal of the characterisation was two-fold: to confirm the quality of the mechanochemically synthesised samples, and to provide a comprehensive and carefully curated benchmark dataset for future experimental and computational studies.

The synthetic method used to obtain halide perovskite materials significantly influences their properties, such as elemental composition, purity and structural stability (Fig. 1b). As we demonstrate here through TGA, solution-grown perovskite crystals incorporate significant amounts of solvent into their crystal structure, evidenced by a low-temperature mass loss (Fig. 1d), in addition to impurity peaks visible in PXRD (Fig. S3, ESI†). Moreover, varying solute–solvent interactions frequently result in a hampered control over the elemental stoichiometries of perovskite products.^28^ Mechanosynthesis instead overcomes the challenges associated with alternative synthetic methods by avoiding issues such as elevated temperatures, complex purification steps as well as limited solubility of precursors, which prevented us from accessing the Cl-rich compositions using solution growth (Fig. 2c). A shaker mill (Fig. 1c) accelerates metal balls in the reaction chamber, crushing and mixing the precursors. Fine-ground precursor powders display greater ion diffusion and enhanced chemical reactivity, leading to a reaction sustained with the constant supply of mechanical energy. The mechanosynthetic method presented here allowed for the synthesis of a complete compositional series of CsPb(Cl_*x*_Br_1−*x*_)_3_ solid-solution crystals (Fig. 1e) in gram-scale amounts (Table S1, ESI†).

**Fig. 2:**
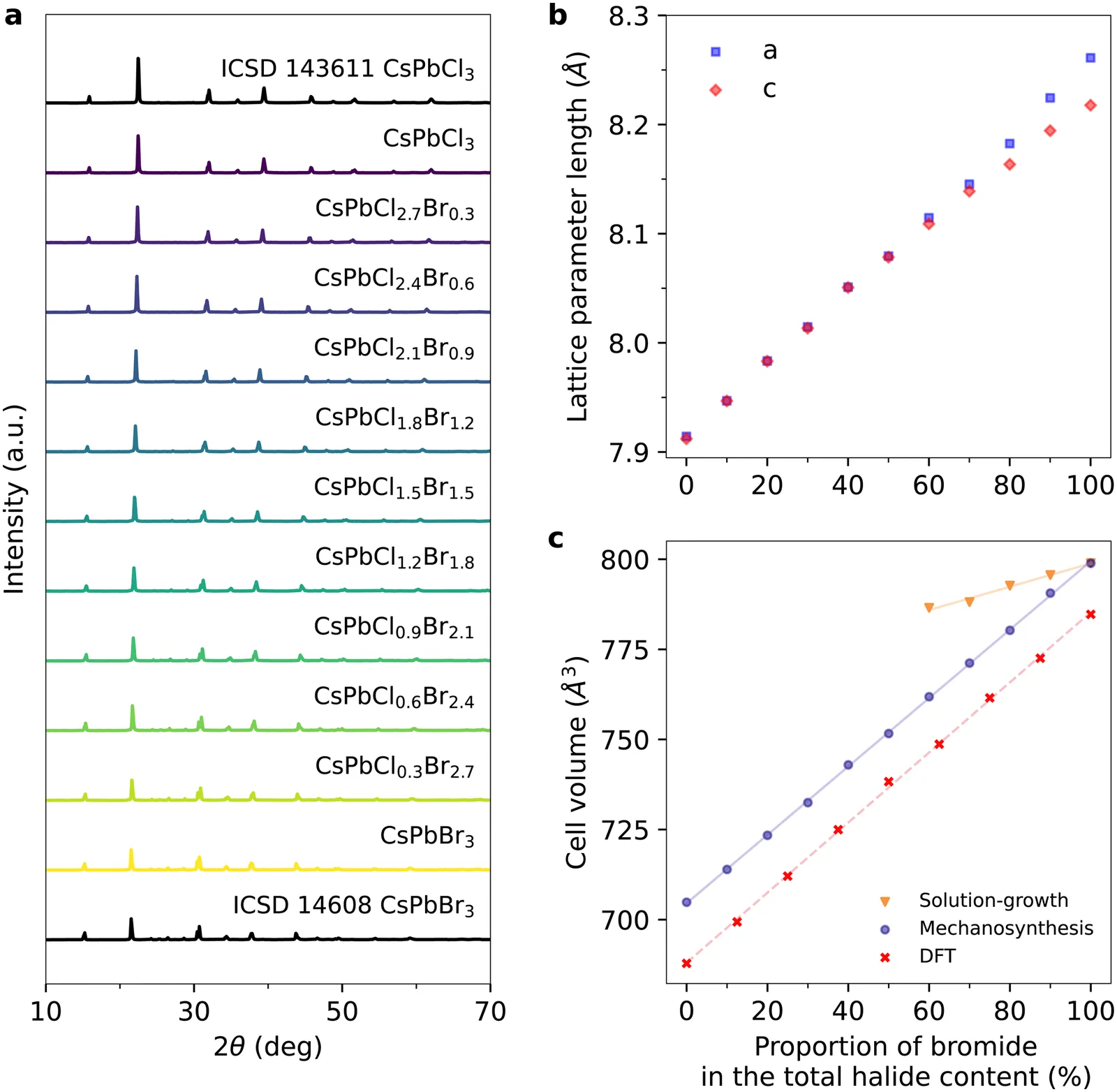
(a) Powder XRD patterns of the mechanosynthesised CsPb(Cl_*x*_Br_1−*x*_)_3_ samples. (b) Dependence of lattice parameters *a* and *c* on halide content in the mechanosynthesised powders. (c) Dependence of the cell volume on halide content in CsPb(Cl_*x*_Br_1−*x*_)_3_ samples prepared by solution growth and mechanosynthesis, respectively. DFT-relaxed cell volumes for a set of special quasirandom structures^11^ (SQS) are shown for comparison and confirm the Vegard-like behaviour; the overall shift is due to a typical slight overbinding of the particular DFT functional.

Powder X-ray diffraction (PXRD) patterns can be qualitatively compared with published data to assess the phase composition and purity of the synthesised materials. Quantitative analysis using Rietveld refinement yields lattice parameters of the crystal structure, indicating the extent of halide mixing according to Vegard's law.^29^ Firstly, the PXRD patterns of mechanosynthesised samples (Fig. 2a) show a good qualitative match with reference data for the single-halide compositions CsPbCl_3_ (ICSD 143611^12^) and CsPbBr_3_ (ICSD 14608^30^), indicating a successful formation of perovskites with *Pnma* space group. This qualitative match was quantified by performing a Rietveld refinement against data taken from the CsPbCl_3_ CIF file (ICSD 143611^12^). The occupancies of Br and Cl in the file were modified to represent the halide mixing ratios in the experimental samples. No unassigned peaks indicating other phases are present in the diffraction patterns of any of the mechanosynthesised samples. Secondly, PXRD patterns of all samples, regardless of the synthetic route, show a progression towards higher diffraction angles: as the chloride ratio increases, the unit-cell parameters decrease. The trend was quantified by plotting the resulting cell volumes of samples at various mixing ratios (Fig. 2c). Vegard's law predicts the material's parameters to vary proportionally with compositional changes. The literature values for the unit cells of CsPbCl_3_^12^ and CsPbBr_3_^30^ are 700.21 Å^3^ and 792.86 Å^3^ respectively, consistent with the end points of our mechanosynthesised series, and a linear trend between them with varied *x* is seen as expected, consistent with first-principles DFT data from earlier studies^31–33^ and the present work (see ESI† for computational details). In contrast, the plot for solution-grown crystals indicates a deviation between the nominal and actual halide compositions, due to challenges in stoichiometic control of this synthetic method. This highlights, together with the above mentioned TGA results, the higher quality of powder over solution-grown crystals which is typically overlooked, and led us to choose the former for building our data library.

As the content of chloride increases, the *a* and *c* lattice parameters become closer in value (Fig. 2b), leading to an almost tetragonal crystal structure and a corresponding diffraction pattern obtained in a conventional powder diffraction measurement. Historically, the similarity between measured lattice parameters led to an ambiguity in the assignment of the space group of CsPbCl_3_, later resolved by synchrotron X-ray powder diffraction performed by Linaburg *et al.*^34^ The study confirmed that CsPbCl_3_ crystallises in the orthorhombic *Pnma* space group with *a* and *c* parameters of 7.90193(1) Å and 7.89928(1) Å respectively, closely matching the values obtained in this work. The gradual trend in lattice parameters and powder diffraction patterns indicates that the *Pnma* space group is common to all halide mixing ratios in the CsPb(Cl_*x*_Br_1−*x*_)_3_ perovskite series.

Next, optical characterisation in the form of photoluminescence (PL) and reflectance was performed to investigate the electronic structure of the series. Reflectance spectroscopy is the standard approach to calculate the optical band gap of halide perovskite powders, providing a convenient point of comparison to literature to ensure the identity of the synthesised material. Moreover, PL spectroscopy offers a pathway to investigate the radiative decay of excited states. Therefore, changes in the band gap caused by altering the halide composition can be used to assess the degree of actual halide mixing, as well as the purity and homogeneity of the samples.

The analysis of diffuse reflectance data reveals that all samples show a clear absorbance onset that progresses gradually towards higher energies as the proportion of chloride increases (Fig. 3b). The absorbance onset was quantified by the Tauc method^35^ to obtain the trend of optical band gap shifting with composition (Fig. 3a and Fig. S1, ESI†). The reflectance spectra (shown as *F*(*R*) plots in Fig. 3b) feature a regular peak–shoulder structure at all halide mixing ratios. The extracted optical band gap energies of CsPbCl_3_ and CsPbBr_3_, 2.93 ± 0.05 eV and 2.30 ± 0.05 eV, respectively, agree very well with previously reported values.^36–38^ We carried out DFT computations at the HSE06^39^ level for the end members of the series and constructed an SQS^11,13^ model for *x* = 0.5, obtaining results in good agreement with our experimental data and with previous computational work.^32,33^ Moreover, the optical band gap energies show the expected linear dependence (*R*^2^ = 0.998) on the proportion of bromide content, in agreement with Vegard's law. Therefore, the comparison with literature data supports the argument for the formation of the mixed-anion halide perovskites, while the continuous tuning of electronic properties implies a close match between the nominal and actual halide compositions.

**Fig. 3:**
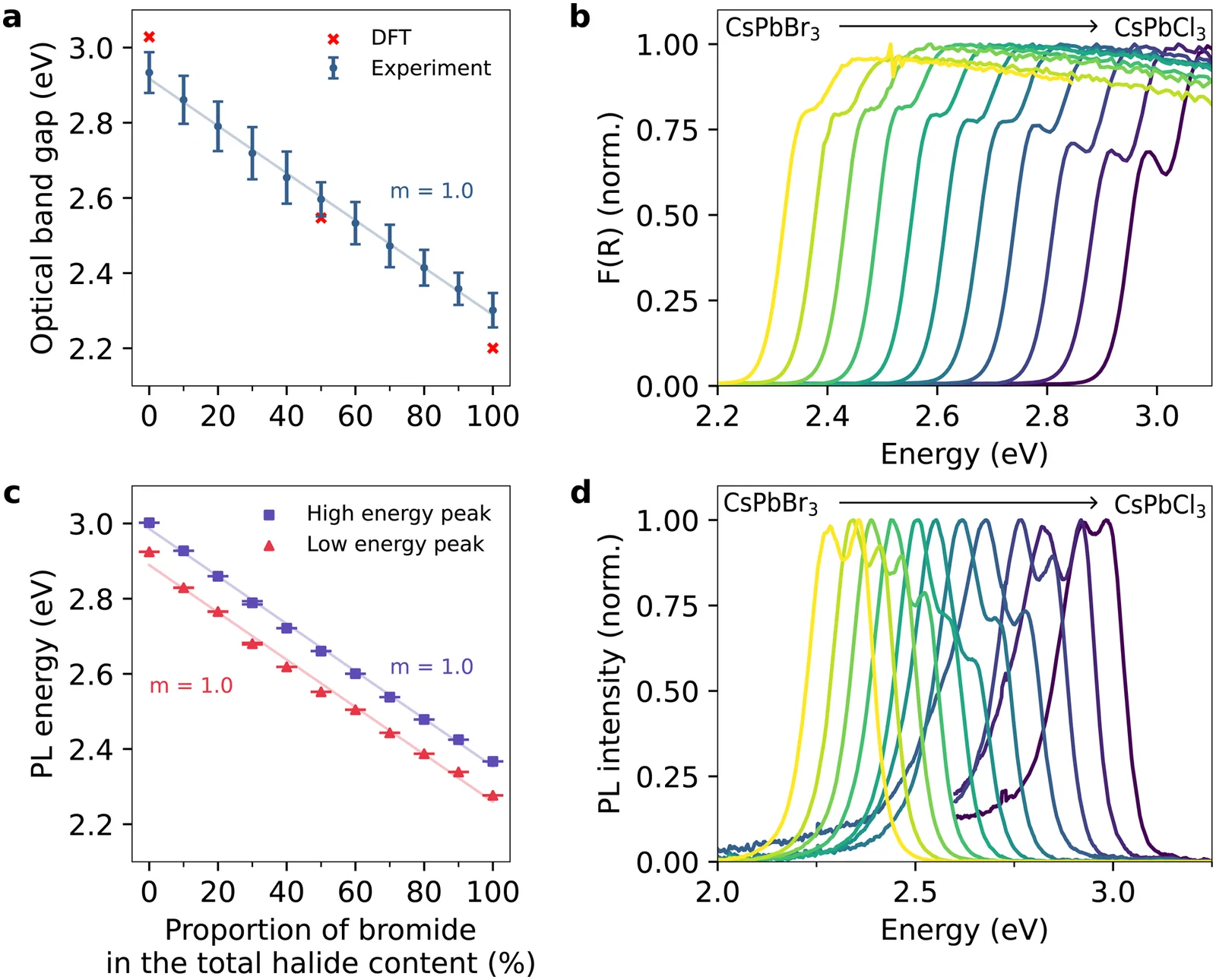
Optical properties. (a) Dependence of the optical band gap determined from diffuse reflectance on halide composition of CsPb(Cl_*x*_Br_1−*x*_)_3_ perovskites. Computed optical band gaps based on DFT at the HSE06^39^ level (see ESI† for details). (b) Normalised absorbance spectra of a series of CsPb(Cl_*x*_Br_1−*x*_)_3_ obtained from diffuse reflectance measurements with an integrating sphere. (c) Dependence of the photoluminescence (PL) peak energies on halide composition of CsPb(Cl_*x*_Br_1−*x*_)_3_ perovskites. (d) Normalised PL spectra of CsPb(Cl_*x*_Br_1−*x*_)_3_.

Similar to the reflectance experiments, PL spectra also revealed a consistent peak progression with changing anion composition (Fig. 3c). A regular progression towards higher energies with increasing chloride content was found, analogously to the optical band gap values from reflectance. In addition to this general trend, the PL measurements show a double peak emission for all halide ratios. The wavelength of both lower and higher-energy peaks shifts linearly with halide composition, in agreement with Vegard's law (Fig. 3d). Although halide perovskites of type CsPbX_3_ are direct band gap semiconductors a double-peak emission is commonly reported in the literature for both large single crystals and powder samples.^40,41^ Such a double-peak structure should not be taken as a sign of inhomogeneity or impurities, and is instead attributed to a self-absorption effect, whose impact is intensified by strong internal reflections.^41–44^ Crucially, the optical band gap and absorption onset for single-halide perovskites are consistent with reports for bulk perovskites throughout the literature.^40,45^

In conclusion, we have identified mechanosynthesis as a suitable method to give access to a complete compositional series of high-quality CsPb(Cl_*x*_Br_1−*x*_)_3_ solid-solution crystals, avoiding solvent incorporation and miscibility gaps, and enabling also the synthesis of select, hitherto unreported compositions. The obtained samples show the expected structural and optical properties, also compared with DFT calculations, confirming purity and homogeneity. The resulting consistent data library, including crystallographic and optoelectronic data, can serve as a benchmark for future experimental and theoretical modelling studies of halide perovskite materials.

This work was performed in part at the Harvard University Center for Nanoscale Systems (CNS); a member of the National Nanotechnology Coordinated Infrastructure Network (NNCI), which is supported by the National Science Foundation under NSF award no. ECCS-2025158. B. B. acknowledges funding from the EPSRC Centre for Doctoral Training in Inorganic Chemistry for Future Manufacturing (OxICFM), EP/S023828/1. The authors are grateful for the assistance of Dr Shao-Liang Zheng, Dr Nicholas Colella and Dr Arthur McClelland with the X-ray, TGA and UV-Vis data acquisition respectively.

## Conflicts of interest

There are no conflicts to declare.

## Supplementary Material

CC-061-D5CC00735F-s001

## Data Availability

Data accompanying this work are available in the ESI† associated with this publication and online on Zenodo at DOI: 10.5281/zenodo.14187372. Crystallographic data were deposited at CCDC under accession numbers CCDC 2423993 to CCDC 2424003 and can be obtained from www.ccdc.cam.ac.uk/structures.
