# Supplementary material for: An experimental data library for the full CsPb(ClxBr1−x)3 compositional series
Source: Chem Commun (Camb). 2025 Mar 31;61(33):6146–9. doi: 10.1039/d5cc00735f (PMC13559777; doi:10.1039/d5cc00735f)
Supplement: Supplementary file 1 [file CC-061-D5CC00735F-s001.pdf]

Supplementary Information (SI) for Chemical Communications.
This journal is © The Royal Society of Chemistry 2025

Supplementary Information†

An experimental data library for the full $CsPb(Cl_xBr_{1-x})_3$ compositional series

Kinga O. Mastej, Bodoo Batnaran, Antti-Pekka M. Reponen, Zachary A. VanOrman, Kal Banger, Michael A. Hayward, Volker L. Deringer, Sascha Feldmann

†Experimental and computational data were also uploaded to Zenodo at DOI: 10.5281/zenodo.14187372. Crystallographic data were deposited at CCDC under accession numbers CCDC 2423993 to CCDC 2424003 and can be obtained from www.ccdc.cam.ac.uk/structures.

Synthetic Methods

Mechanosynthesis

Mechanosynthesis in a ball mill was used to synthesise $CsPb(Cl_xBr_{1-x})_3$ compositional series. The ball mill accelerates metal balls in the reaction chamber, crushing and mixing the precursors. A series of 11 $CsPb(Cl_xBr_{1-x})_3$ samples, spanning the halide ratio with $0 \leq x \leq 1$ at $x = 0.1$ step, was prepared stoichiometrically, according to the equation:

$$\mathrm{xCsCl} + (1-\mathrm{x})\mathrm{CsBr} + \mathrm{xPbCl_2} + (1-\mathrm{x})\mathrm{PbBr_2} \rightarrow \mathrm{CsPb(Cl_xBr_{1-x})_3}$$

CsCl (99.999%), CsBr (99.999%), $PbBr_2$ (99.999%) and $PbCl_2$ (99.999%) were purchased from Sigma-Aldrich and used without further purification. Precise proportions of reagents for different halide compositions are presented in Table S1. A 100 mL milling container was filled with the reagents and 3 stainless steel balls of 10 mm diameter with the total weight of 12.45 g. The balls to precursor mass ratio was 8.3:1. The milling container was sealed in the nitrogen atmosphere before the reaction start to avoid oxidation. The reactions were performed in Retsch MM 500 nano mixer mill at 30 Hz for 5 hours. After the ball-milling, samples were opened and stored in a glovebox before further characterisation.

	CsCl	$PbCl_2$	CsBr	$PbBr_2$
$CsPb(Cl_{1.0},Br_{0.0})_3$	0.5657 g (3.36 mmol)	0.9344 g (3.36 mmol)	0.0000 g (0 mmol)	0.0000 g (0 mmol)
$CsPb(Cl_{0.9},Br_{0.1})_3$	0.5091 g (3.02 mmol)	0.8410 g (3.02 mmol)	0.0715 g (0.31 mmol)	0.1233 g (0.31 mmol)
$CsPb(Cl_{0.8},Br_{0.2})_3$	0.4526 g (2.69 mmol)	0.7476 g (2.69 mmol)	0.1430 g (0.65 mmol)	0.2466 g (0.65 mmol)
$CsPb(Cl_{0.7},Br_{0.3})_3$	0.3960 g (2.35 mmol)	0.6541 g (2.35 mmol)	0.2145 g (0.99 mmol)	0.3699 g (0.99 mmol)
$CsPb(Cl_{0.6},Br_{0.4})_3$	0.3394 g (2.01 mmol)	0.5607 g (2.01 mmol)	0.2860 g (1.33 mmol)	0.4933 g (1.33 mmol)
$CsPb(Cl_{0.5},Br_{0.5})_3$	0.2828 g (1.67 mmol)	0.4672 g (1.67 mmol)	0.3575 g (1.67 mmol)	0.6166 g (1.67 mmol)
$CsPb(Cl_{0.4},Br_{0.6})_3$	0.2263 g (1.33 mmol)	0.3738 g (1.33 mmol)	0.4290 g (2.01 mmol)	0.7399 g (2.01 mmol)
$CsPb(Cl_{0.3},Br_{0.7})_3$	0.1697 g (0.99 mmol)	0.2803 g (0.99 mmol)	0.5005 g (2.35 mmol)	0.8632 g (2.35 mmol)
$CsPb(Cl_{0.2},Br_{0.8})_3$	0.1131 g (0.65 mmol)	0.1869 g (0.65 mmol)	0.5720 g (2.69 mmol)	0.9865 g (2.69 mmol)
$CsPb(Cl_{0.1},Br_{0.9})_3$	0.0566 g (0.31 mmol)	0.0934 g (0.31 mmol)	0.6435 g (3.02 mmol)	1.1098 g (3.02 mmol)
$CsPb(Cl_{0.0},Br_{1.0})_3$	0.0000 g (0 mmol)	0.0000 g (0 mmol)	0.7150 g (3.36 mmol)	1.2332 g (3.36 mmol)

Table S1. Amounts of reagents used, in grams and moles, for the preparation of a series of $CsPb(Cl_xBr_{1-x})_3$ solid-solutions through mechanosynthesis.

Solution growth

Inverse temperature crystallisation method (ITC) was used to grow $CsPb(Cl_xBr_{1-x})_3$ single crystals from solution. This technique leverages the decreasing solubility of halide perovskites as the temperature increases. The synthetic procedure developed by Wang et al. was followed precisely[1].

Characterisation Methods

Powder X-Ray Diffraction (PXRD)

Powder XRD measurements were performed on a Bruker D2 PHASER diffractometer equipped with a 300 W generator (30.0 kV, 10.0 mA) and a Cu anode with 1.54184 Å wavelength at 0.4° step. The samples were mounted on a silicon holder with a 10 mm wide and 0.2 mm deep cavity. Rietveld refinement was carried out using the TOPAS v6 software[7,8].

Thermogravimetric Analysis (TGA)

TGA measurements were performed on a Discovery 550 manufactured by TA Instruments with a high-temperature platinum pan. The procedure consisted of an isothermal step, where the sample was held at 25°C for 1 min to equilibrate the temperature, and a ramp to 500°C at 20°C per minute. The experiment was performed in a nitrogen atmosphere.

UV-Vis reflectance

Diffuse reflectance spectra of powders were measured using a Cary 7000 UV-VIS-NIR spectrophotometer. Measurements were carried out in an integrating sphere, with a Spectralon disc used as a reference sample.

Reflectance values were used to estimate absorbance *via* the Kubelka-Munk transform[9], which assumes reflectance from a layer of semi-infinite thickness (R_∞):

$$F(R_\infty) = \frac{(1 - R_\infty)^2}{2R_\infty} = \frac{k}{s}$$

From which the quantity $\frac{k}{s}$ is obtained, containing a scattering coefficient s and an absorption coefficient for semi-infinite layers given by k. Under the assumption that the scattering coefficient is not strongly wavelength dependent and the sample sufficiently thick that the measured reflectivity $R \approx R_\infty$, we can approximate $F(R)$ to be linearly proportional to the sample absorption coefficient.

To approximate band gap values from reflectance data we use the Tauc method[10], where the absorption coefficient α at the absorption edge is assumed to be a function of photon energy $h\nu$ and optical band gap Eg as:

$$(\alpha h\nu)^2 \propto h\nu - Eg$$

With the exponent of 2 resulting from a direct band gap[11]. Under the assumptions listed before, a plot of $(F(R)h\nu)^2$ as a function of photon energy $h\nu$ should be linear near the absorption edge, and a line interpolated from this linear portion will then have an x-intersect at the optical band gap.

Tauc plots with associated linear interpolants are presented in Figure S1. Raw reflectance data are presented in Figure S2.

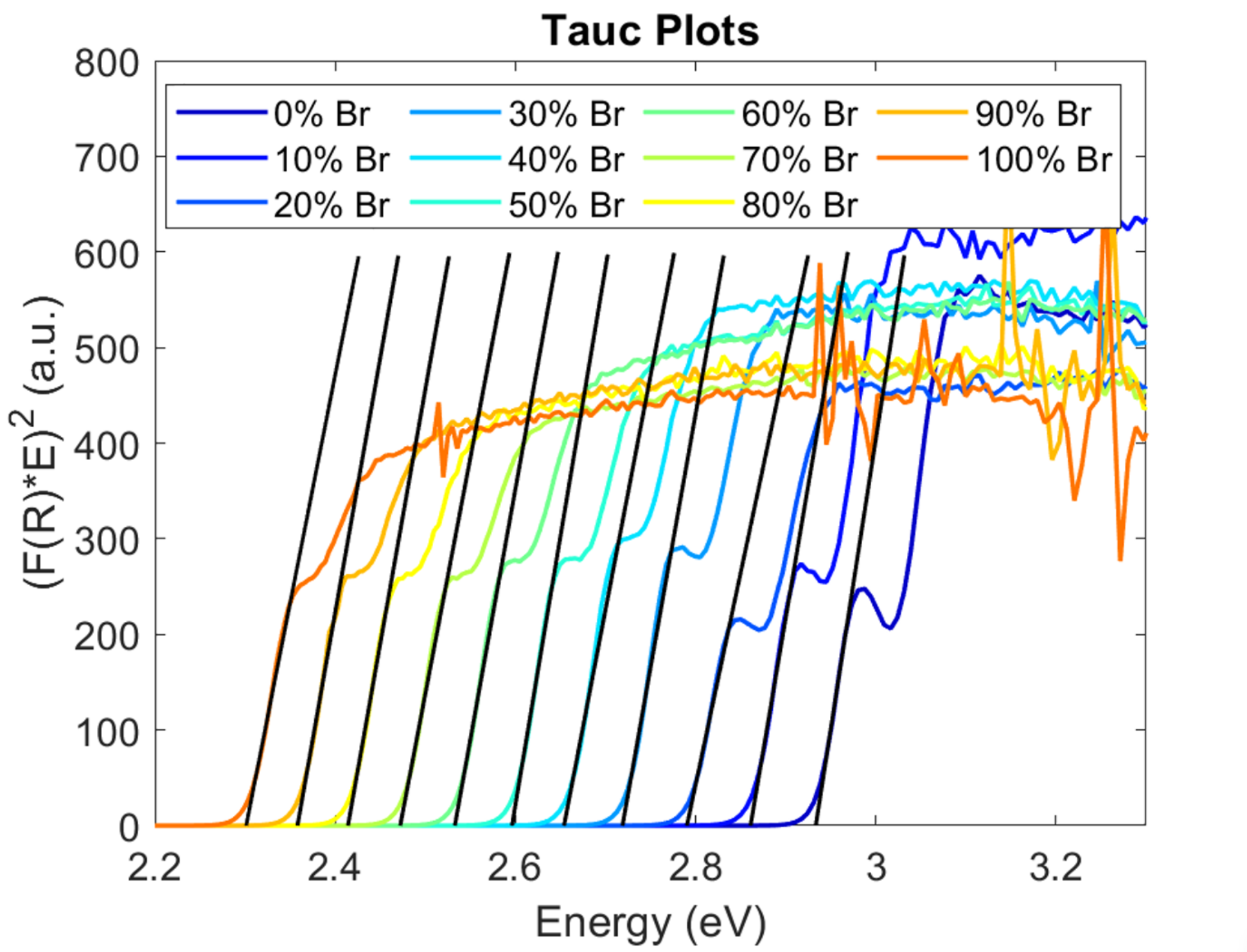

Figure S1. Tauc plots with the scaled reflectance-derived absorption estimate $(F(R)h\nu)^2$ for the series of synthesized $CsPb(Br_xCl_{100\%-x})_3$ powders and linear interpolations to the absorption onset edges (black lines) from which optical band gaps are estimated.

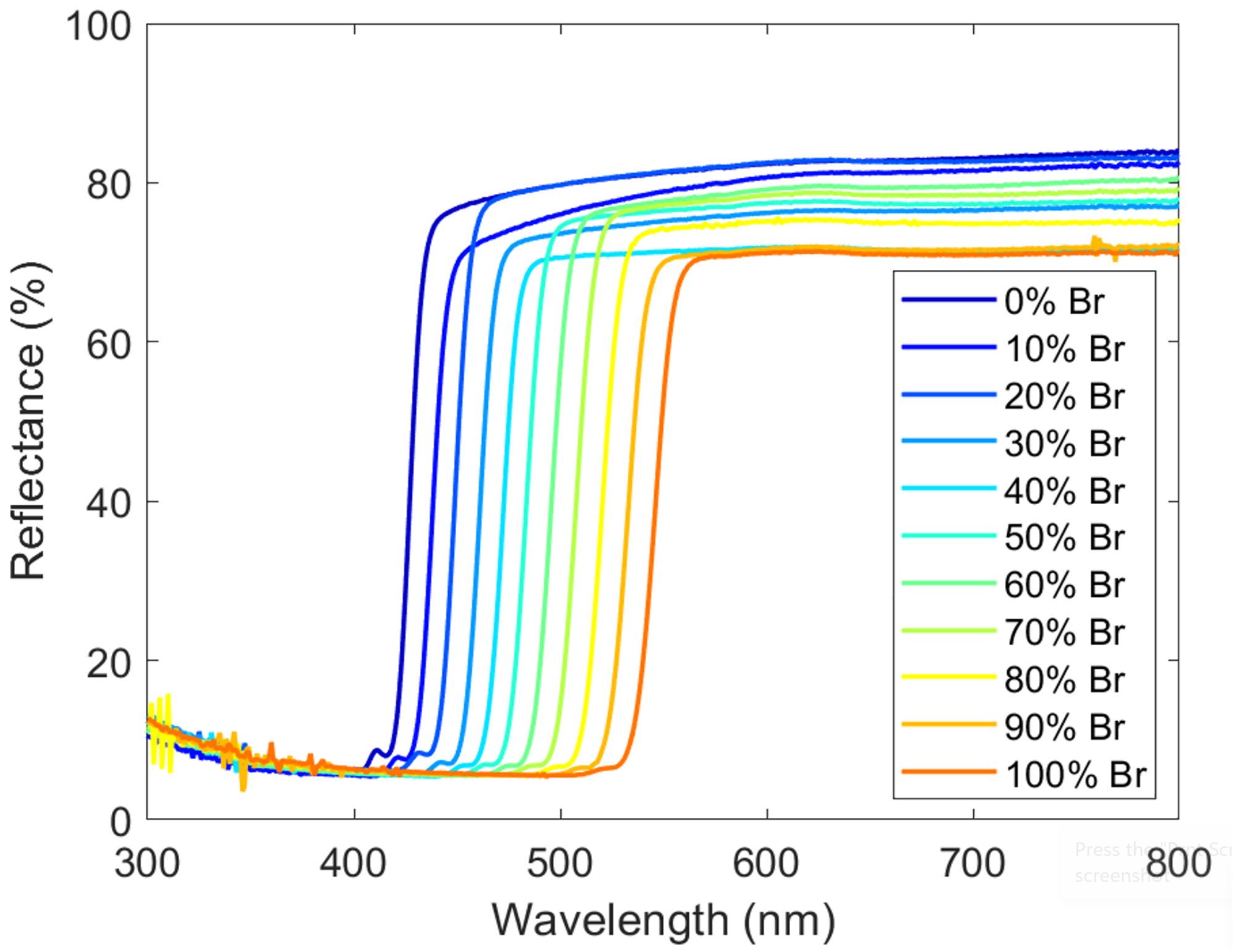

Figure S2. Raw diffuse reflectance data for the series of synthesized $CsPb(Br_xCl_{100\%-x})_3$ powders.

Photoluminescence spectroscopy (PL)

PL spectra were measured with a ThorLabs Compact Spectrometer CCS200. Samples were excited with a 343 nm pulse laser (50 kHz repetition rate, 200 fs pulse width) at 3.5 mW average power. A 380 nm longpass filter was used to block excitation scatter. To mount samples, powders were encapsulated between two coverslips using hardened epoxy resin. Some sample degradation during measurements was apparent, especially for materials with higher Cl content, which contributed to the low-energy tails observed in these materials.

Computational methods

To study the mixed compositions, 80-atom special quasi-random structures[12] (SQS) were generated from an experimental $CsPbCl_3$ CIF file[2,13] using ASE[14] and icet[15] tools. A Monte-Carlo simulated annealing algorithm was employed to find the structure with the closest objective function to the random anion distribution. The associated calculations were accomplished using plane-wave density functional theory (DFT) within CASTEP[16]. On-the-fly generated norm-conserving pseudopotentials were used. All geometry optimisations used the PBEsol[17] functional with a plane-wave cutoff energy of 1400 eV and *k*-point mesh of 2x3x2 for the supercell and *k*-point mesh of 7x5x7 for the end members. Using the optimised geometries, electronic density of states calculations were carried out with the HSE06[18] functional with a plane-wave cutoff energy of 300 eV and Γ-point only for the x=0.5 SQS and 3x3x3 *k*-point mesh for the end members.

Tabulated Data

Optical Band Gap

CsBr/(CsBr + CsCl) (%)	Optical band gap (eV)
0	2.9334(543)
10	2.8610(638)
20	2.7904(659)
30	2.7189(693)
40	2.6540(692)
50	2.5962(454)
60	2.5329(562)
70	2.4723(565)
80	2.4143(476)
90	2.3582(427)
100	2.3013(457)

Table S2. Optical band gaps of a series of $CsPb(Cl_xBr_{1-x})_3$ solid-solutions obtained *via* mechanosynthesis.

Photoluminescence Peaks

CsBr/(CsBr + CsCl) (%)	High energy peak (eV)	Low energy peak (eV)
0	3.0020(1)	2.9246(2)
10	2.9273(1)	2.8291(2)
20	2.8596(2)	2.7656(3)
30	2.7890(42)	2.6802(22)
40	2.7215(1)	2.6185(1)
50	2.6605(1)	2.5521(1)
60	2.6004(1)	2.5046(1)
70	2.5380(1)	2.4431(1)
80	2.4785(1)	2.3874(1)
90	2.4248(1)	2.3390(1)
100	2.3670(1)	2.2768(1)

Table S3. Photoluminescence peak positions of a series of $CsPb(Cl_xBr_{1-x})_3$ solid-solutions obtained *via* mechanosynthesis.

Experimental Lattice Parameters

CsBr/(CsBr + CsCl) (%)	a (Å)	b (Å)	c (Å)	Volume (Å^3)
0	7.914(2)	11.2557(5)	7.912(2)	704.8(3)
10	7.946(3)	11.3048(5)	7.946(3)	713.9(4)
20	7.983(3)	11.3504(6)	7.983(3)	723.3(5)
30	8.014(3)	11.4048(6)	8.013(3)	732.4(4)
40	8.050(5)	11.4621(8)	8.050(5)	742.9(7)
50	8.079(5)	11.5159(8)	8.078(5)	751.6(7)
60	8.114(2)	11.5780(7)	8.108(2)	761.8(2)
70	8.145(1)	11.632(1)	8.138(1)	771.1(1)
80	8.182(1)	11.681(7)	8.163(1)	780.2(1)
90	8.2243(5)	11.7312(7)	8.1943(5)	790.60(9)
100	8.2610(5)	11.7680(6)	8.2176(5)	798.89(8)

Table S4. Experimental lattice parameters of a series of $CsPb(Cl_xBr_{1-x})_3$ solid-solutions obtained *via* mechanosynthesis.

DFT Lattice Parameters

CsBr/(CsBr + CsCl) (%)	a (Å)	b (Å)	c (Å)	Volume (Å^3)
0	7.7709	11.2558	7.9122	704.82
12.5	7.9469	11.304	7.9468	713.92
25	7.9834	11.3504	7.9832	723.40
37.5	8.0146	11.4048	8.0134	732.47
50	8.0510	11.4621	8.0507	742.93
62.5	8.0795	11.5159	8.0785	751.64
75	8.1146	11.5781	8.1090	761.85
87.5	8.1453	11.6329	8.1389	771.19
100	8.1825	11.6812	8.1635	780.28

Table S5. DFT-calculated lattice parameters of a series of $CsPb(Cl_xBr_{1-x})_3$ solid-solutions.

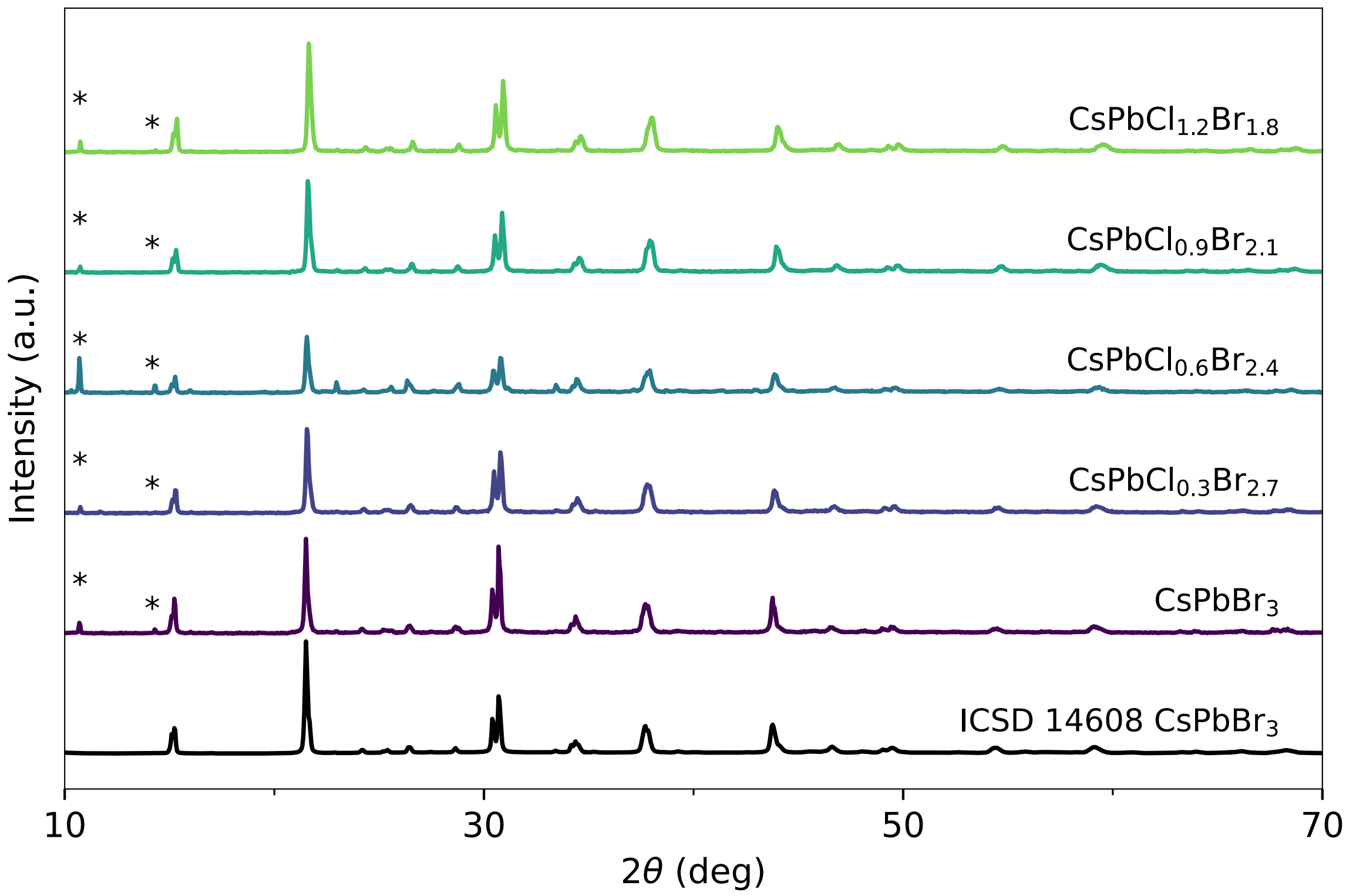

Figure S3. Powder XRD patterns of the solution-grown $CsPb(Cl_xBr_{1-x})_3$ samples. Impurity peaks are denoted by asterisks.

Solution-grown crystals exhibit impurity peaks at 10.8° and 14.3° originating from $PbBr_2(DMSO)_2$ complex[19]. The synthetic method applied[1] aimed to avoid the formation of a CsBr-rich Cs_4PbBr_6 impurity phase by carrying out the synthesis in the presence of excess $PbBr_2$. This macroscopic disparity was assumed to yield a precise control of the CsBr:$PbBr_2$ ratio on the molecular scale *via* solvent engineering of DMSO, DMF and CyOH proportions. The PXRD data of solution-grown perovskites suggests instead that the excess $PbBr_2$ formed a complex with the DMSO solvent. DMSO has a high coordination ability with Pb^{2+}, forming Pb—O bonds of 2.386[20] Å, which explains why $PbBr_2(DMSO)_2$ forms preferentially to pure $PbBr_2$ or an alternative $PbBr_2$DMF complex (Pb—O bond length of 2.431[20] Å) in the presence of both solvents.

Determination of the chemical composition of the mechanosynthesised samples: Methodology

Obtained samples show the expected structural and optical fingerprints, also compared with DFT calculations, confirming the composition of the samples.

PXRD patterns of mechanosynthesised samples (Fig. 2a) show a good match with reference data for the single-halide compositions $CsPbCl_3$ (ICSD 143611[2]) and $CsPbBr_3$ (ICSD 14608[3]), indicating a successful formation of these perovskites in the anticipated *Pnma* space group. This finding was further quantitatively confirmed by performing a Rietveld refinement against the theoretical structures of mixed-halide perovskites, at all synthesised mixing ratios. No unassigned peaks indicating other phases are present in the diffraction patterns of any of the mechanosynthesised samples. Moreover, Vegard's law predicts that materials' parameters vary proportionally with compositional changes. Indeed, the literature values for the unit cell volumes of $CsPbCl_3$[2] and $CsPbBr_3$[3] are 700.21 Å^3 and 792.86 Å^3 respectively, consistent with the end points of our mechanosynthesised series, and a gradual trend in unit cell volumes (as well as optical band gaps and photoluminescence peak energies) with R^2 = 0.99 indicates achievement of the mixed-ratio compositions in the $CsPb(Cl_xBr_{1-x})_3$ perovskite series.

Next, optical characterisation in the form of photoluminescence and reflectance spectroscopy was performed to confirm the electronic variations within the series, which depend on the mixing ratio. Reflectance spectroscopy was chosen as the standard approach to calculate the optical band gap of halide perovskite powders, providing a convenient point of comparison to literature and ensuring the identity of the synthesised material. On the other hand, PL spectroscopy offers a pathway to investigate the radiative decay of excited states and potential presence of any impurities. Therefore, changes in the band gap, caused by altering the halide composition, can be used to assess the degree of halide mixing, as well as the purity and homogeneity of the samples. The extracted optical band gap energies of $CsPbCl_3$ and $CsPbBr_3$, 2.93±0.05 eV and 2.30±0.05 eV, respectively, agree very well with previously reported values[4–6] and show linear dependence on the proportion of bromide content, again in agreement with Vegard's law.

References

1 F. Wang, H. Zhang, Q. Sun, A. B. Hafsia, Z. Chen, B. Zhang, Y. Xu and W. Jie, *Crystal Growth & Design*, 2020, **20**, 1638–1645.
2 M. Szafrański, A. Katrusiak and K. Ståhl, *Journal of Materials Chemistry A*, 2021, **9**, 10769–10779.
3 C. A. López, C. Abia, M. C. Alvarez-Galván, B.-K. Hong, M. V. Martínez-Huerta, F. Serrano-Sánchez, F. Carrascoso, A. Castellanos-Gómez, M. T. Fernández-Díaz and J. A. Alonso, *ACS Omega*, 2020, **5**, 5931–5938.
4 M. Stefanski, B. Bondzior, A. Basinski, M. Ptak, B. Lou and C.-G. Ma, *Inorganic Chemistry Frontiers*, 2024, **11**, 2626–2633.
5 J. Gong, H. Zhong, C. Gao, J. Peng, X. Liu, Q. Lin, G. Fang, S. Yuan, Z. Zhang and X. Xiao, *Advanced Science*, 2022, **9**, 2201554.
6 H. Zhang, X. Liu, J. Dong, H. Yu, C. Zhou, B. Zhang, Y. Xu and W. Jie, *Crystal Growth & Design*, 2017, **17**, 6426–6431.
7 A. A. Coelho, *Journal of Applied Crystallography*, 2018, **51**, 210–218.
8 Bruker (2017). TOPAS. Version 6.0. Bruker AXS, Karlsruhe, Germany.
9 Kubelka, P. & Munk, F., *Zeitschrift für Technische Physik*, 1931, **12**, 593–601.
10 J. Tauc, R. Grigorovici and A. Vancu, *Physica Status Solidi B*, 1966, **15**, 627–637.
11 J. I. Pankove, *Optical Processes in Semiconductors*, Courier Corporation, 1975.
12 A. Zunger, S.-H. Wei, L. G. Ferreira and J. E. Bernard, *Physical Review Letters*, 1990, **65**, 353–356.
13 D. Zagorac, H. Müller, S. Ruehl, J. Zagorac and S. Rehme, *Journal of Applied Crystallography*, 2019, **52**, 918–925.
14 A. H. Larsen, J. J. Mortensen, J. Blomqvist, I. E. Castelli, R. Christensen, M. Dułak, J. Friis, M. N. Groves, B. Hammer, C. Hargus, E. D. Hermes, P. C. Jennings, P. B. Jensen, J. Kermode, J. R. Kitchin, E. L. Kolsbjerg, J. Kubal, K. Kaasbjerg, S. Lysgaard, J. B. Maronsson, T. Maxson, T. Olsen, L. Pastewka, A. Peterson, C. Rostgaard, J. Schiøtz, O. Schütt, M. Strange, K. S. Thygesen, T. Vegge, L. Vilhelmsen, M. Walter, Z. Zeng and K. W. Jacobsen, *Journal of Physics: Condensed Matter*, 2017, **29**, 273002.
15 M. Ångqvist, W. A. Muñoz, J. M. Rahm, E. Fransson, C. Durniak, P. Rozyczko, T. H. Rod and P. Erhart, *Advanced Theory and Simulations*, 2019, **2**, 1900015.
16 S. J. Clark, M. D. Segall, C. J. Pickard, P. J. Hasnip, M. I. J. Probert, K. Refson and M. C. Payne, *Zeitschrift für Kristallographie*, 2005, **220**, 567–570.
17 J. P. Perdew, A. Ruzsinszky, G. I. Csonka, O. A. Vydrov, G. E. Scuseria, L. A. Constantin, X. Zhou and K. Burke, *Physical Review Letters*, 2008, **100**, 136406.
18 J. Heyd, G. E. Scuseria and M. Ernzerhof, *The Journal of Chemical Physics*, 2003, **118**, 8207–8215.
19 W. Wang, Y. Wu, D. Wang and T. Zhang, *ACS Omega*, 2019, **4**, 19641–19646.
20 Y. Wu, A. Islam, X. Yang, C. Qin, J. Liu, K. Zhang, W. Peng and L. Han, *Energy Environ. Sci.*, 2014, **7**, 2934–2938.
